# The influence of hydrothermal fatigue on the clinically relevant functional properties of conventional glass-ionomer cements

**DOI:** 10.1038/s41598-023-35880-4

**Published:** 2023-05-30

**Authors:** Magdalena Łępicka, Agata Maria Niewczas, Magdalena Urszula Rodziewicz, Konrad Pikuła, Paweł Kordos, Tomasz Gredes, Krzysztof Jan Kurzydłowski

**Affiliations:** 1 Faculty of Mechanical Engineering, Institute of Mechanical Engineering, Bialystok University of Technology, Wiejska 45C St., 15-352 Bialystok, Poland; 2 Department of Conservative Dentistry with Endodontics, Medical University of Lublin, W. Chodzki 6, 20-093 Lublin, Poland; 3 Institute of Transport, Combustion Engines and Ecology, Lublin University of Technology, Nadbystrzycka 36, 20-618 Lublin, Poland; 4 Department of Orthodontics, Technische Universität Dresden, Carl Gustav Carus Campus, Fetscherstr. 74, 01307 Dresden, Germany; 5 Department of Orthodontics and Temporomandibular Disorders, Poznan University of Medical Sciences, Bukowska 70, 60-812 Poznan, Poland

**Keywords:** Engineering, Materials science

## Abstract

During their everyday service, the restorative dental materials are subjected to temperature changes which can be viewed as intensive in the context of the highest allowed temperatures for these materials. In this work, the effect of hydrothermal fatigue on the in vitro tribological performance, compression strength, microhardness, and surface roughness of glass-ionomer cements was studied. Samples of 3 commercially available cements were divided into the reference (aged 14 days) and thermocycled (20,000 cycles; 5–55 °C) groups. The results obtained show that functional properties of the specimens subjected to thermal fatigue significantly differ from the literature data on the cements aged at constant temperatures. The effect of hydrothermal fatigue on the functional properties of cements is discussed in the context of processes induced by exposure to variable temperatures.

## Introduction

In modern dentistry, reliable, safe, and environment-friendly materials for permanent dental fillings are still sought. The mercury-based amalgam materials are to be phased-out in EU countries by 2030^1^, while resin-based composites (RBCs) and glass-ionomer cements (GICs) are considered appropriate alternatives. Their advantages include, among others, good aesthetic effect, lack of mercury in composition, as well as no risk of metallic corrosion. During their service, both types of restorative materials, RBCs and GICs, are exposed to a wide range of biological, chemical, and physical degradation factors, including mechanical, hydrothermal, and tribological loads^2^. The degradation processes of the restoratives lead to microfractures and cracks^2,3^, as well as severe tribological wear of both restoration and the opposing teeth^2^. The volumetric changes of the restoration due to cohesive shrinkage as well as oral thermal changes cause a microleakage gap between the restorative and the tooth^4^. Those damages are known to promote colonization of the oral bacteria and biofilm associated with recurrent caries and hypersensitivity of the teeth^2^, what ultimately leads to the placement of the repair restoration.

It was assessed that after 2000s, 58% of the total dental placement was related to the replacement of the currently existing restoration due to failure^5^. The data collected between 2000 and 2019 shows that in RBC restorations, bulk fractures and wear accounted for 70% of all reported failures^6^. On the other hand, the overall survival rate of GIC restorations after 6 years of service was 80%^6^. These statistics pay attention to the insufficient longevity of dental restorations, what is directly associated with increased health care cost caused by the recurrent dental interventions.

Among the main factors influencing longevity of dental restorations, resistance to wear and fracture were listed^2,6,7^. Therefore, to approximate the operating conditions of a restorative material, various types of tests are proposed, including randomized clinical trials and in vitro testing^8^. While clinical trials are still considered the best method to evaluate the quality and longevity of dental materials, there are many factors which limit the trials applications, including their time and cost consumption^8^. Moreover, due to large variability in operators, different patient compliance, etc., standardization and replicability of clinical trials are difficult to achieve^8^. Therefore, in vitro testing, which makes it possible to replicate, to some extent, the oral cavity environment and the stresses which are borne both by the teeth and the restorations, is routinely proposed to assess e.g. strength^8^ or tribological^9,10^ performance of restoratives. However, there is some criticism regarding applicability and robustness of the in vitro tests recommended by the International Organization for Standardization (ISO)^11–13^. For example, the ISO tests do not take into account the long-term impact of the oral environment on the maturation processes of GICs. On the other hand, one of the factors which is inevitable during service of a dental restoration is the hydrothermal fatigue^14^. It concerns clinically relevant properties of the restoratives, such as their surface microhardness, compressive strength, or resistance to wear^15^.

It is estimated that every day, human oral environment is exposed to action of 20 to 50 thermal cycles^16^. Those are responsible for generating temperature-dependent variable stresses within the polymer matrix of both RBCs and GICs. While polymers are poor conductors of heat, each cycle of heating and subsequent cooling contributes to emergence of thermal stresses at the restorative material’s surface. Moreover, the mismatch in coefficient of thermal expansion between the restorative and the tooth contributes to the thermal strain of a restoration^17^. Therefore, to obtain reliable information on the projected long-term behaviour of a restorative dental material in an in vitro test, simulation of hydrothermal fatigue is essential.

To simulate hydrothermal fatigue of dental materials, numerous protocols were developed^8,14,16,18^. One of the first recommendations was included in the 1994’s ISO TR 11405 standard^18^, where thermocycling regimen consisting of 500 cycles in water between 5 and 55 °C was suggested^8^. On the other hand, in the protocol presented by Gale and Darvell in 1999^16^, 10,000 thermal cycles in a temperature interval of 35–15–35–45 °C were proposed to simulate 1 year of an in vivo service of a restoration. However, up to date, a great variability in testing methods was seen, including dwell time, temperature range, number of temperatures in the cycle, number of cycles, and others^8,16^. For example, in the works published since 1998, the most popular aging testing method is thermocycling in the temperature range from 5 to 55 °C^8^. However, it has to be noted that, in contrast to resin-based restorative^8,19^ and bonding^8,20^ materials, the literature on the effect of hydrothermal aging on the properties and performance of glass-ionomers is still scarce^21–24^. This might be due to the fact that for many years, glass-ionomers were considered suitable mostly for temporary restorations or the non-stress bearing sites^25^. Nevertheless, there are some indications where GICs are used as permanent filling materials, e.g. in atraumatic restorative treatment (ART)^26,27^ which is often performed in children^27,28^, and people with disabilities^28^. GICs use in the case of children is highly recommended, because compared to adults, in juvenile patients the occlusal forces are relatively small^27^.

Taking into account the current state of knowledge and the achievements in the clinical applications of glass ionomer cements, the authors of this work conducted an in vitro study on the effect of hydrothermal fatigue on selected functional properties of conventional glass-ionomer cements. The clinically relevant properties, such as: microhardness, compressive strength, tribological performance, and surface roughness, were discussed. The experimental material is enriched with statistical analysis of data, as well as microscopic analyses.

## Materials and methods

### Materials

Three commercially available self-curing, hand-mixed glass-ionomer cements were used in the study: Ketac Universal, Ketac Molar Easymix (both produced by 3M ESPE, ST. Paul, USA), and Riva Self Cure (SDI Ltd., Bayswater, Australia). For all tested materials, A3 shade was used. All packages of a given type of material had the same LOT number.

According to the manufacturer’s data^29^, Ketac Universal (KU) is impermeable to X-rays. It is used to fill cavities in primary and permanent teeth, including, among others, stress bearing sites, cervical areas, Class V, and multi-surface restorations. Moreover, it can be used for tooth core build-up prior to crown placement, fissure sealing, and as a lining for single- and multi-surface composite fillings.

The second tested material was Ketac Molar Easymix (KME)^30^. Its indications for use include semi-permanent Class III and V restorations of primary and permanent teeth, and single- and multi-surface restorations. Additionally, it can be used for restorations of primary teeth, as a liner under composite and amalgam restorations, as well as for cervical fillings without cosmetic priorities. It is impermeable to X-rays.

The third tested material was Riva Self Cure (RSC)^31^. This glass-ionomer cement is used to fill Class I, II and V cavities in permanent teeth, as well as for deciduous teeth restorations. Moreover, it can successfully be used for core build-up, dentine replacement, as well as in temporary fillings, and minimally invasive dentistry. Like the previous two, it is characterized by X-ray permeability. The chemical compositions of all tested materials are shown in Table S1 (Supplementary Information).

It should be emphasized that manufacturers are not obliged to include in their Safety Data Sheets the substances which are deemed harmless. For this reason, the chemical composition of liquid and powder ingredients presented in Supplementary Table S1 shall not be considered fully reliable. For example, in case of KME cement, the manufacturer does not clearly specify the full chemical composition of the liquid^32^. Nevertheless, some evident differences in compositions can be seen, e.g. in tartaric acid content. Usually, 5 to 10 wt% of this acid is used in the liquid ingredient. The tartaric acid is intended to extend the working time and improve setting stage of the material^33^. In addition, powder ingredient of one of the tested cements (KU) does not contain polyacrylic acid.

### Sample preparation

The glass-ionomer samples were prepared according to ISO 9917-1^34^ and manufacturers’ recommendations. GICs were agglutinated in room temperature, by one professional dental operator. Samples were obtained by hand agglutination with a metal spatula on a mixing pad, using the powder and liquid ingredients in ratios recommended by the manufacturers, that is, one level spoonful of powder to one drop of aqueous polyacid solution. The cement was placed in the mould immediately after agglutination. After a setting time of 1 h, each sample was transferred to a laboratory incubator set at 37 °C. As a storage medium, deionized water was used. For the measurements of microhardness, surface roughness, friction, and volumetric wear, disc-shaped samples with a diameter of 10 mm and a thickness of 2 mm were made. On the other hand, cylindrical specimens with diameter of 4 mm and a height of 6 mm were used for compressive strength test, as recommended in ISO 9917-1^34^.

In total, 6 series of samples were manufactured, and divided into 2 groups. The first one contains reference samples, i.e. not subjected to thermal loads (abbreviations: RSC—Riva Self Cure, KU—Ketac Universal, KME—Ketac Molar Easymix). Those were aged for 14 days in distilled water under constant temperature (37 °C). The latter are the samples subjected to artificial hydrothermal fatigue regimen (abbreviations with suffix TC, e.g. RSC_TC). A summary of the fabricated samples and the number of measurements performed is provided in Supplementary Fig. S1 (online).

### Methods

#### Hydrothermal fatigue

Accelerated hydrothermal fatigue protocol was applied using a laboratory simulator described in^14,35^. The device consisted of a hydraulic module and a microprocessor-based control system. During aging, each sample was placed in a test vessel which was alternately filled with deionized water at 55 °C or 5 °C. One thermal cycle consisted of the following stages: (a) filling the measuring vessel with heated/cooled water for 10 s; (b) sample dwelling in the heated/cooled water for 30 s; and (c) draining the measuring vessel from the heated/cooled water for 10 s. The dwell time was selected according to related prior publications^8^. For each sample of series 4–6 (Supplementary Fig. S1), 20,000 hydrothermal cycles were done. In the previous works, it was stated that approximately 10,000 thermal cycles correspond to 1 year of in vivo placement of a dental restoration^8,16^.

#### Microhardness

Microhardness measurements were carried out according to the Vickers method using a microhardness tester MicVision VH-1 (Sinowon; Dong Guan, China) and a measurement procedure similar to^36^. A load of 200 g was applied, which was equal to a normal force of 1.96 N. For each sample series, n = 16 measurements were taken. The hardness was calculated using the formula:

1 $$HV = 0.102 \times \frac{{2{\text{F}}sin68^{^\circ } }}{{d^{2} }}$$

where *HV*—Vickers hardness [N/mm^2^], F—normal force [1.96 N], *d*—average diagonal of the indentation [mm].

#### Compressive strength

An MTS 322 dynamic testing machine (MTS Systems; Eden Prairie, USA) was used for compressive strength tests and the requirements of ISO 9917-1^34^ were considered. To ensure uniform load of a sample, each specimen was positioned in a central axis of the compression jaw disk. The test conditions were as follows: a force increment speed of 0.5 N/s, a crosshead movement speed of 0.005 mm/s, and a sampling frequency of 0.02 Hz. For each sample series, n = 5 tests were performed. The compressive strength was calculated using the formula:

2 $$R_{c} = \frac{{F_{n} }}{A}$$

where *R*_*c*_—compressive strength [MPa], *F*_*n*_—ormal force to fracture [N], *A*—surface area of the cross-section perpendicular to the long axis of the sample [mm^2^].

#### Surface roughness

Using confocal laser microscope, n = 16 line surface roughness measurements were carried out for each series of samples. The sampling length (λ_c_) equalled 800 µm, which was the width of the optical field of view obtained with a 20 × lens. It was then taken 5 times for averaging, so that a single evaluation length equalled 4 mm. On the other hand, λ_s_ was set at 2.5 µm in order to exceed the optical resolution of the used lens, and to be equal at least three times of the value of data sampling interval. The following roughness parameters were recorded: the arithmetic average surface roughness Ra, and the mean peak to valley height Rz.

#### Tribological performance

Wear resistance tests were conducted on a biotribometer (UMT-2, Bruker; Billerica, USA). The experimental conditions were selected as for mastication and oral environment lubrication. A commercial mucin-rich artificial saliva solution was used as a lubricant (Kserostemin, Aflofarm; Poland). The disc-shaped test sample with a diameter of 10 mm and a height of 2 mm was moving in a reciprocating motion. Teeth shift by of 0.5 mm was assumed, which is close to as reported in^37,38^. Therefore, the length of a friction track was fixed at 500 µm (Fig. S2, Supplementary Information). With regards to the normal load, load of 5 N was chosen, which is in the range of values reported elsewhere^36^ for the non-conformal ball-on-disc contacts. As a counter sample, an alumina ball was chosen^39–42^ of 6 mm diameter, following^38^. Lastly, a single chewing cycle is estimated to last between 0.87 and 0.95 s^43^, what corresponds to 1 Hz reciprocating frequency and is in line with other in vitro experiments on restoratives^10^. A single measurement lasted 7200 s, while the frequency of sample sliding was set at 1 Hz. Fluctuations in frictional forces over time were acquired a frequency of 10 Hz. For each sample tested, the value of the average coefficient of friction (COF) was calculated, as well as volumetric wear of material was measured. A total of 30 measurements were done, that is, 5 replications for each sample series tested.

#### Microscopic observations

Volumetric wear measurements were conducted by the non-contact method using a laser confocal microscope (CLSM, LEXT4000, Olympus; Tokyo, Japan). In addition, samples were analysed with the use of an SEM–FIB system (Scios 2 DualBeam SEM–FIB, Thermo Fisher Scientific; Waltham, USA). During the studies of both reference GIC microstructures, and the morphology of the wear tracks, the backscatter electron (T1) *in-lens* detector was used.

#### Statistical analysis

Statistical analysis of research data was done using Statistica 13.3.0 software for Windows 10 (Statistica 13.3.0, Tibco Software; Palo Alto, USA). The assumption of a normal distribution was checked for all variables using the Shapiro–Wilk test. The null hypothesis was sustained for volumetric wear and surface microhardness data. In order to check the homogeneity of the variances within respective groups, Levene’s test was performed. For volumetric wear data, the null hypothesis on the homogeneity of variances was sustained. Therefore, a comparative t test was performed (*p* < 0.05). On the other hand, for microhardness data, the null hypothesis on the homogeneity of variance was rejected. Therefore, F Welch t test was carried out (*p* < 0.05). For COF, surface roughness, and compressive strength data, the null hypothesis of normal distribution was rejected (Shapiro–Wilk test, *p* < 0.05). Thus, differences between respective groups were checked using a U Mann–Whitney test (*p* < 0.05).

## Results

### Microstructural analysis of the as-received glass-ionomer samples

**Figure 1:**
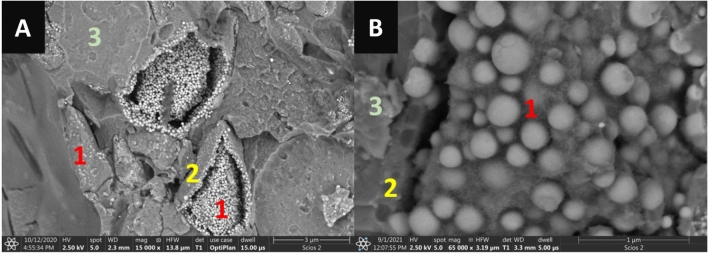
Structure of a representative glass-ionomer cement (3 M Ketac Molar Easymix): 1—non-reacted glass core, 2—silicious hydrogel matrix-glass interphase, 3 – polysalt hydrogel matrix. Note that the glass cores differ in terms of radiopaque nanoparticles content. SEM BSE images, magnifications: 15,000× (**A**) and 65,000× (**B**).

The structure of glass-ionomer cements (Fig. 1A) is obtained in a setting reaction between the fluoroaluminosilicate glass particles and the polyacids. In effect, a microstructure of a conventional GIC (Fig. 1B) features non-reacted glass cores with intermediate siliceous hydrogel layer, embedded in the cross-linked polysalt gel cement matrix^26^. Such a type of a microstructure is found in all tested GICs^44–46^.

### Microhardness

**Figure 2:**
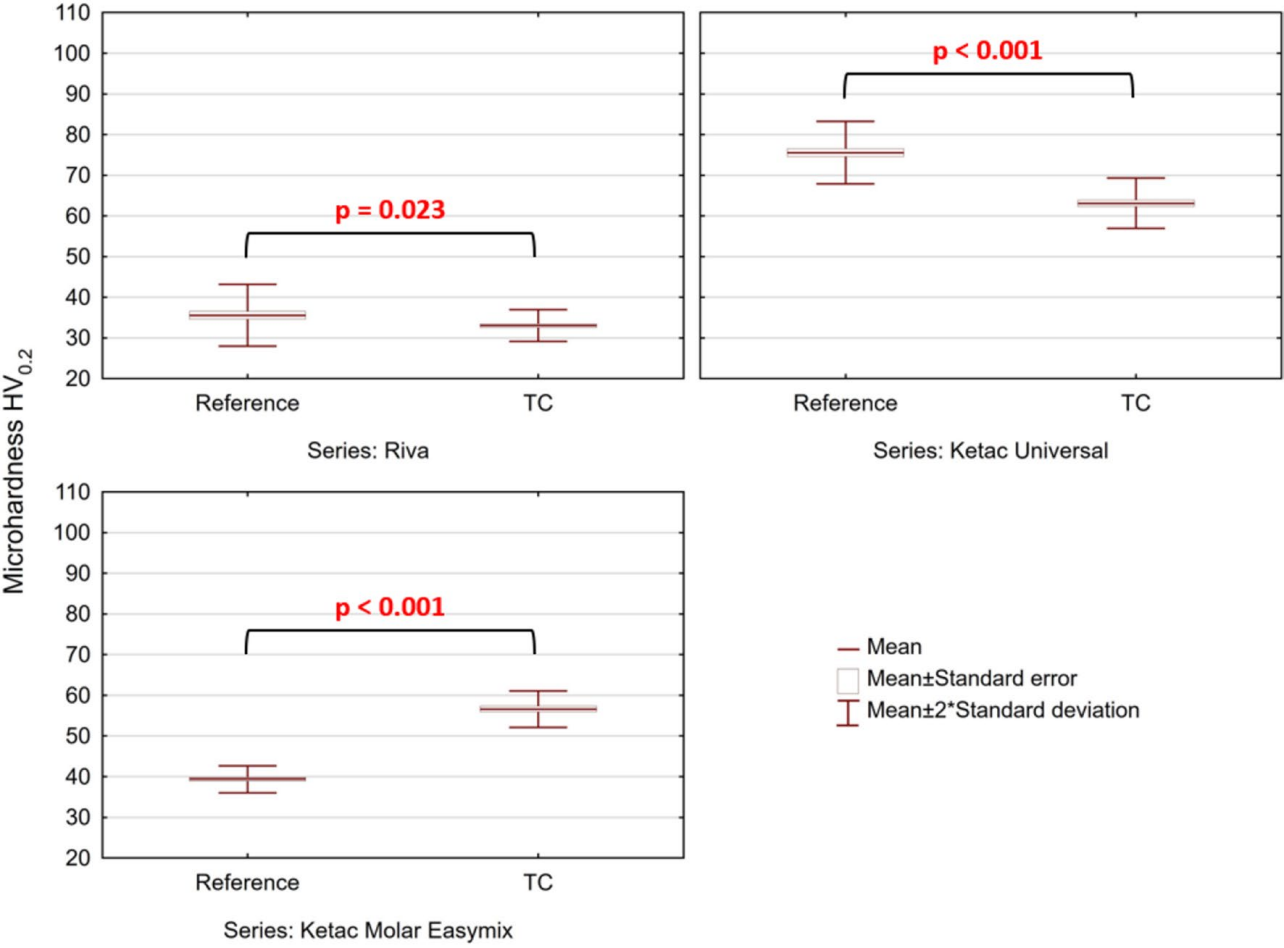
Findings from microhardness tests, conducted for the reference and fatigued specimens (F Welch, *p* < 0.05).

Findings from microhardness measurements taken for both reference and fatigued samples are shown in Fig. 2. For both RSC and KU, after hydrothermal aging, a statistically significant reduction in microhardness was seen (F Welch test, *p* < 0.05). On the other hand, in KME, after thermal fatigue, an increase in microhardness was observed. Moreover, irrespective of the conditioning environment, the greatest microhardness among the tested materials was that of KU, while the lowest of RSC.

### Compression strength

**Figure 3:**
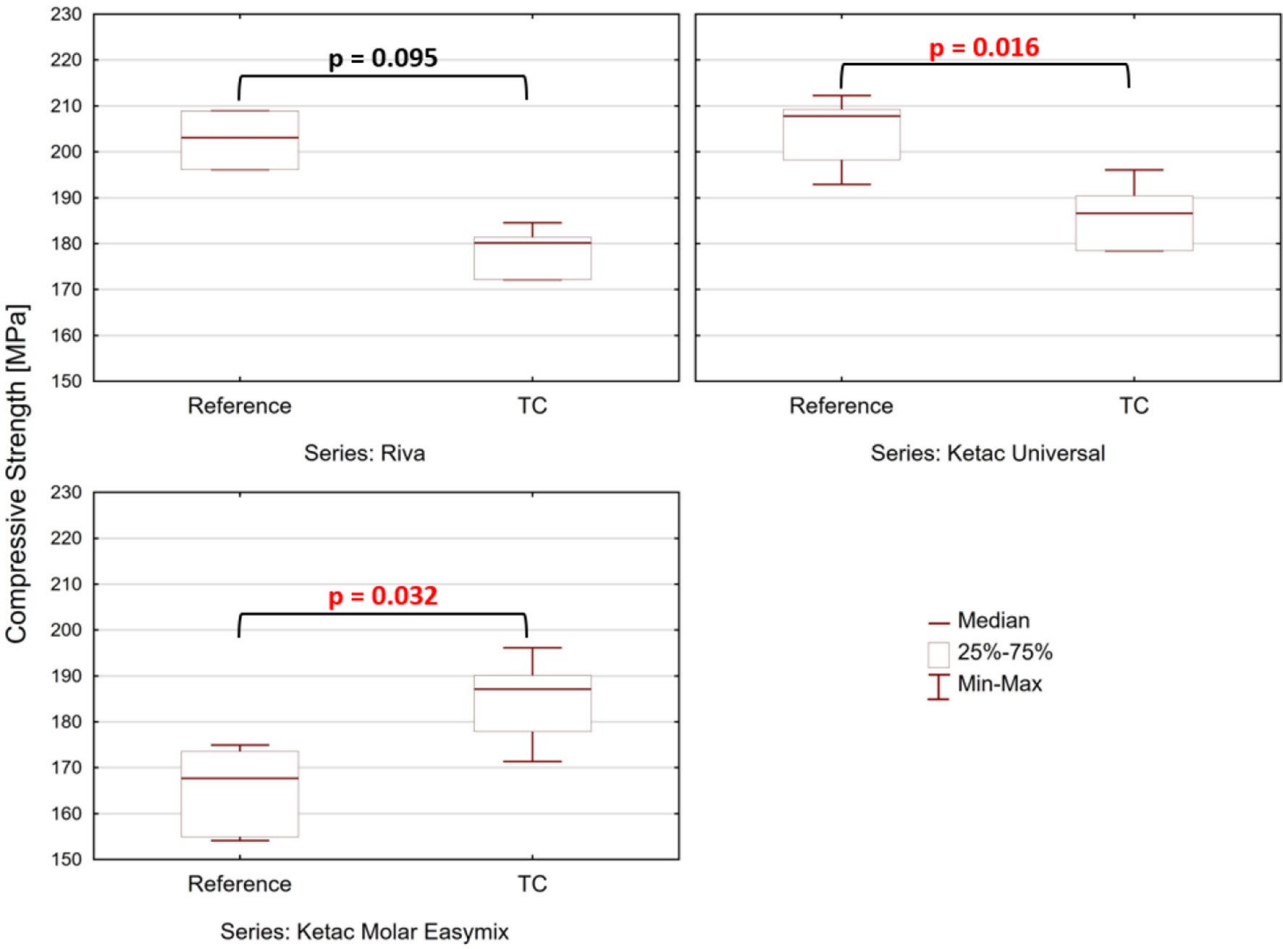
Boxplots presenting distribution in the compressive strength of the reference and the fatigued specimens (U Mann–Whitney, *p* < 0.05).

During the compression strength measurements, stress–strain diagrams were acquired (Supplementary Figs. S3–S5). After calculating the maximum compressive stress for each sample tested, a comparative statistical analysis of the investigated glass-ionomers was carried out. The results are shown in Fig. 3. For KU cement, after hydrothermal aging, a decrease of 8.6% in compressive strength was observed (U Mann–Whitney test, *p* < 0.05; values in the range from 191 to 209 MPa). Simultaneously, a significant increase in compressive strength was found for KME, by 7.4% (in the range from 175 to 188 MPa). Whereas, in the case of RSC, the observed differences were not statistically significant.

### Surface roughness

**Figure 4:**
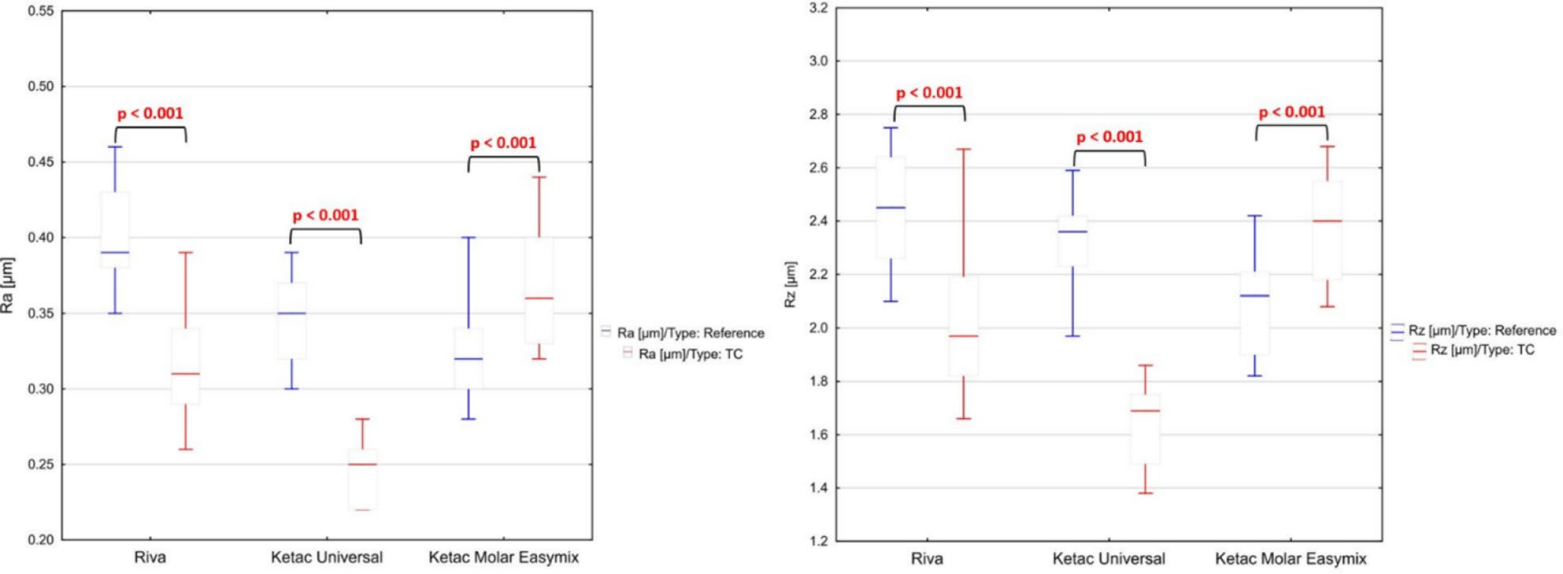
Findings from surface roughness measurements: parameters Ra and Rz (U Mann–Whitney, *p* < 0.05).

Two surface roughness parameters were analysed: Ra, and Rz. For each of the test series, 16 measurements were taken, and the results are shown in boxplots (Fig. 4). Compared to the reference samples, after thermal fatigue, a decrease in the value of the Ra parameter was found for RSC and KU cements, while for KME, an increase in Ra was seen. Analysis of the Rz parameter has also shown similar differences between the tested groups. It was observed that after hydrothermal aging, the Rz parameter decreased for RSC and KU cements. However, an increase was found for KME glass-ionomer.

### Tribological performance

Findings from the friction coefficient measurements are shown in Fig. S6 (Supplementary Information). In each analysed sample series, values of COF were determined from n = 5 measurements. No statistically significant differences in COF between the reference and thermocycled samples were seen. For RSC cement, the scatter of measurement data acquired for fatigued samples was smaller than that of the reference. A similar observation could be done for KME.

**Figure 5:**
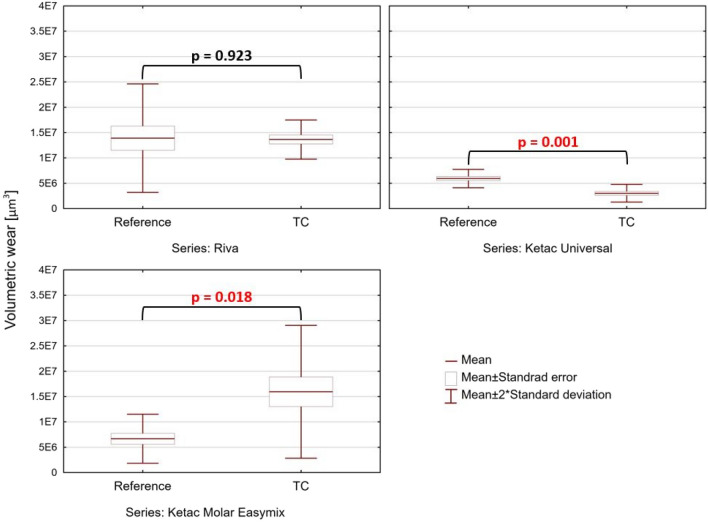
Comparison of the volumetric wear of glass-ionomers: reference versus hydrothermal fatigue (paired t test, *p* < 0.05).

Although the average COF did not change significantly as a result of fatigue (Supplementary Fig. S6), differences were observed in the volumetric wear of the tested materials (Fig. 5). For RSC cement, the wear of specimens remained at a similar level, irrespective of the conditioning method. The differences between both groups were found to be statistically insignificant. However, the wear of KU material subjected to thermal loads decreased significantly, whereas substantial increases in wear were observed for KME (paired t test, *p* < 0.05).

**Figure 6:**
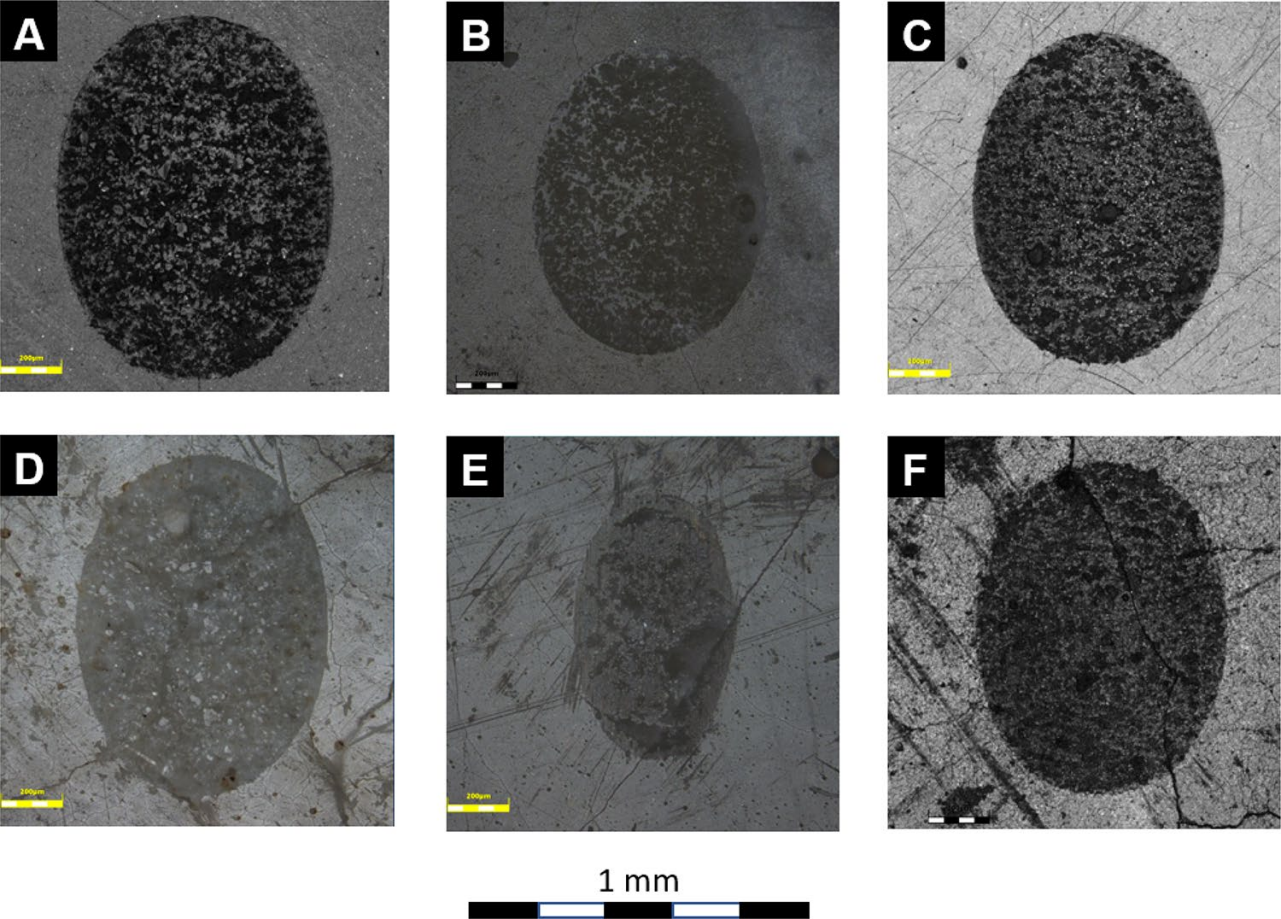
CLSM images of representative friction tracks obtained in the tribological study: (**A**) RSC, (**B**) KU, (**C**) KME, (**D**) RSC_TC, (**E**) KU_TC, (**F**) KME_TC.

The representative two-dimensional CLSM images of the resultant friction tracks are shown in Fig. 6. All reference samples were characterized by a similar shape of the friction tracks—regular and oval (Figs. 6A–C). However, the differences in shape and size of the friction marks of tested materials obtained before and after hydrothermal aging are noteworthy. For KU cement, after thermocycling, the representative wear track became significantly smaller (Fig. 6E)—shorter and narrower. On the other hand, in case of KME, after hydrothermal aging, the wear track became wider (Fig. 6F). For RSC, a shorter and narrower wear track was seen (Fig. 6D). However, after quantifying volumetric wear, only in RSC no differences were seen (Fig. 5).

**Figure 7:**
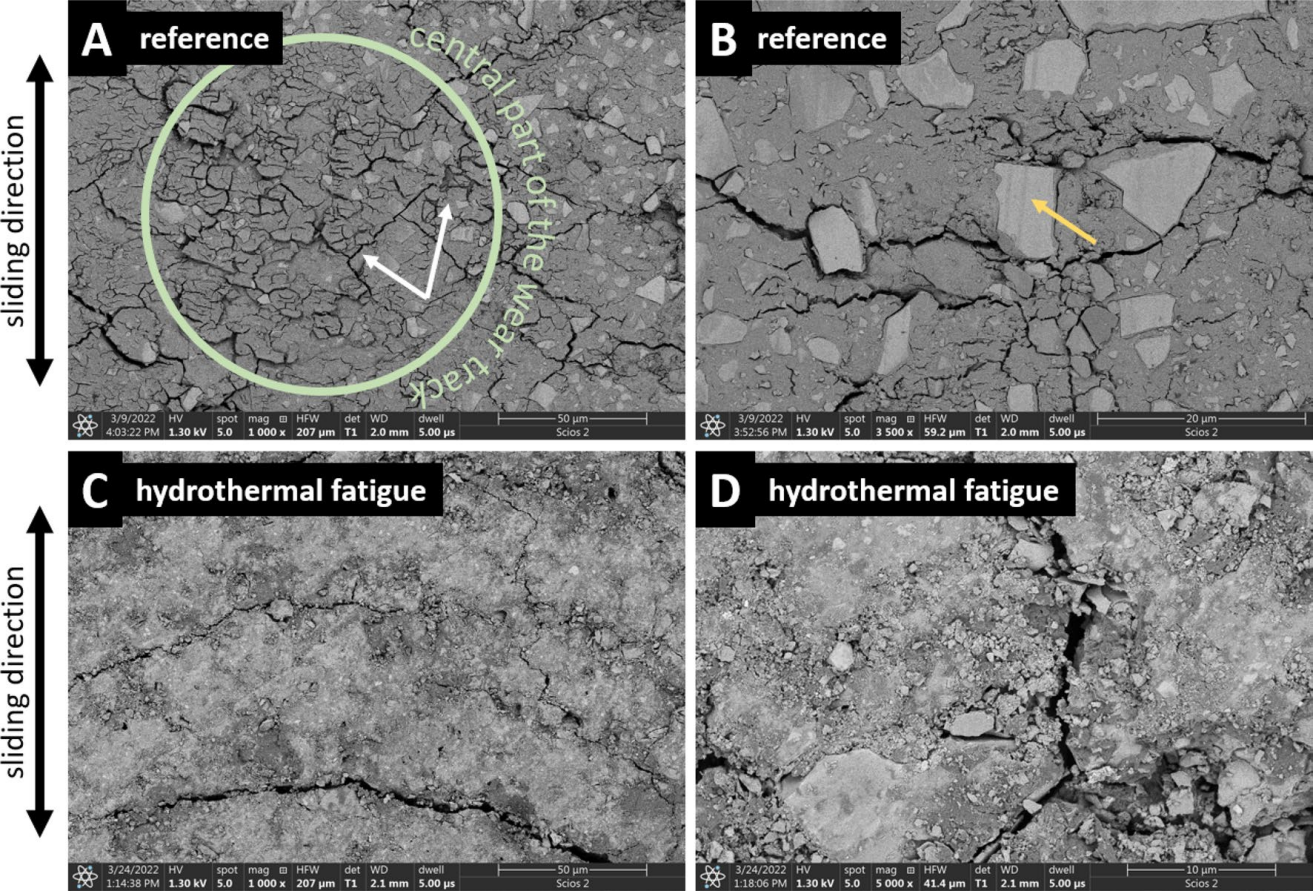
High magnification images of the representative wear tracks obtained for KU samples: (**A**) and (**B**) reference, (**C**) and (**D**) sample subjected to hydrothermal fatigue. SEM BSE images.

To collect information on the dominant wear modes and the wear- and fatigue-induced alterations in the GIC microstructures, high resolution SEM observations were done. In Fig. 7, morphologies of the wear tracks obtained for the reference and thermocycled KU samples are shown. In the reference specimen, two dominant wear modes are present. Tribofilm was formed on the sample surface. It did not adhere well to the frictional track (Fig. 7A). Moreover, abrasive three-body wear of the surface (Fig. 7B) was present. However, after hydrothermal aging, the morphology of the wear track changed. While in Fig. 7A,B no free nano- and microsized wear debris could be seen, in Fig. 7C,D, which represent the fatigued sample, the wear track is covered in free nano- and microparticles. Moreover, signs of tensile cracking of the matrix, were present (Fig. 7C). Nevertheless, after thermocycling, a reduction in volumetric wear of the tested GIC was seen (Fig. 5).

**Figure 8:**
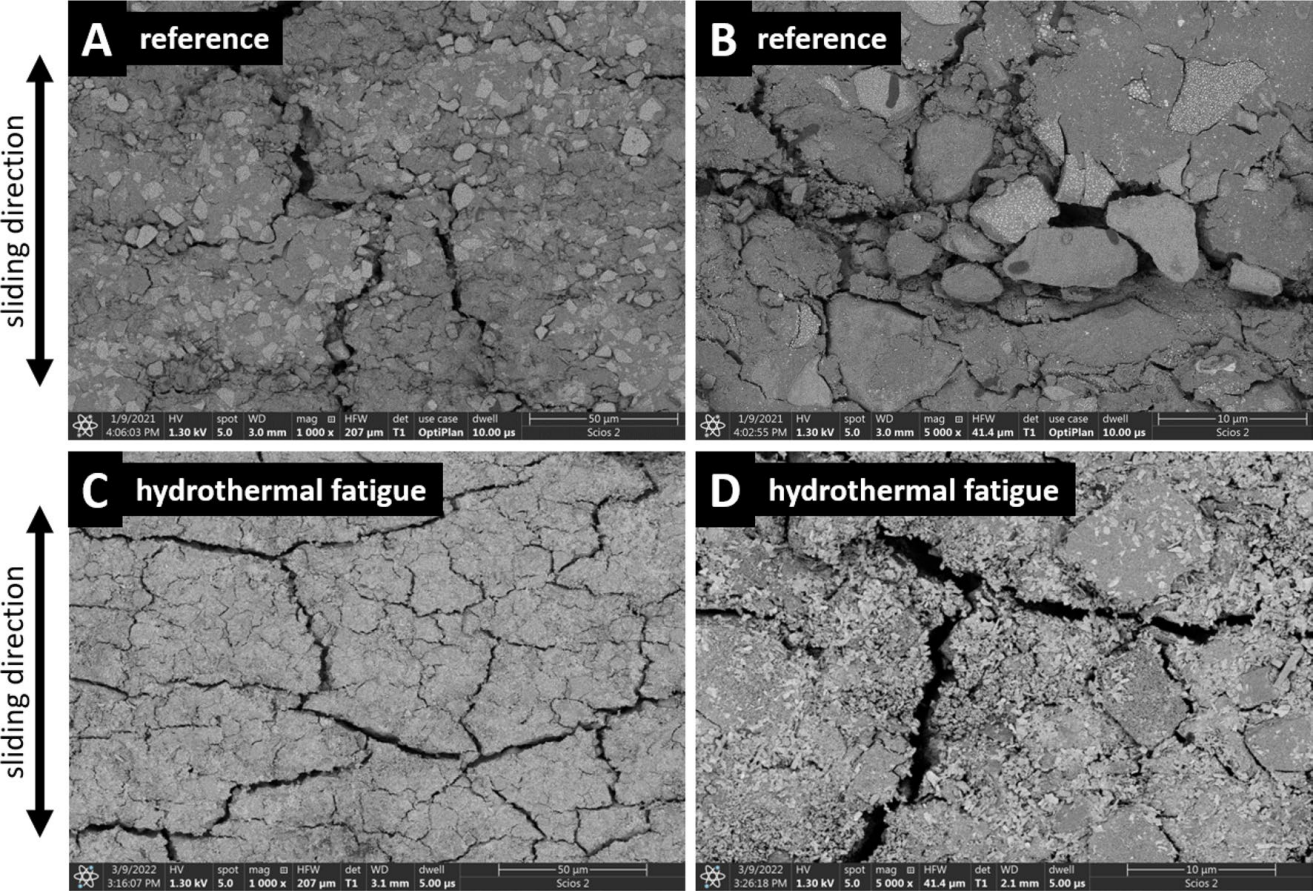
High resolution images of the wear tracks obtained for KME samples: (**A**) and (**B**) reference, (**C**) and (**D**) sample subjected to hydrothermal fatigue. SEM BSE images.

A similar tendency as in KU was seen in KME (Fig. 8). In a reference sample, a tribofilm was formed (Fig. 8A). Some of the glass particles were embedded in the tribofilm, while the others remained free (Fig. 8B). On the other hand, in the fatigued sample (Fig. 8C,D), the elemental contrast between the glass particles and the matrix was poor (Fig. 8C). The wear track was densely covered in micro- and nanometric wear products (Fig. 8D). Moreover, in comparison with the reference (Supplementary Fig. S7A), nanoparticles were not present in the glass particles of fatigued cement (Supplementary Fig. S7B).

The least differences in the wear track morphology between the reference and the fatigued samples were seen for Riva Self Cure (Supplementary Fig. S8). In both cases, two main mechanisms of damage of the glass particles are seen: (a) compressive cracking (Supplementary Fig. S8B,C), and (b) tensile cracking (Supplementary Fig. S8B,D). Moreover, flattening-polishing mechanism was seen in both cases. However, wear performance of the material was not affected by the hydrothermal fatigue regimen (Fig. 5).

## Discussion

In glass-ionomer cements, the ion-leachable glass (base) reacts with the polyalkenoic acid^47^. In the first, rapid (2–6 min) decomposition phase, neutralization between the acidic polyacid solution and the glass particles takes place^26^. As a consequence, ions such as aluminum, fluoride, calcium, or strontium are being released from the glass particle surface^47^. After the release of the ions, the polyacid molecules become ionized and increase their linearity. Thanks to this, the carboxyl groups of the polyacid are more accessible to ions and facilitate their cross-linking in the later stage of gelation^47^, which is followed by cement setting during which the material properties^26,48^, including microhardness and compressive strength, are developed. The finally cured cement will be a composite of cores of unreacted glass particles, encapsulated in silica gel and embedded in a polyacid-salt matrix that binds the components together^26^. As a consequence of the different types of reinforcing particles used in the commercial formulation, the glass particles used within one GIC may differ in their morphology (Fig. 1A). The manufacturers also ensure that their GICs are impervious to X-rays^29–31^. The bright spots visible on the surface of a representative glass particle (Fig. 1B) are the nanoparticles containing radiocontrast agents.

During the maturation stage, which extends to months following the initial setting reaction, various processes take place which result in changes of compressive strength^26,49^, hardness^49–51^, toughness^26,49^, wear performance^49^, and opacity of the restorative^26^. The resultant functional properties of GICs, including their mechanical performance^52–56^, and surface state^57,58^, depend on the conditioning environment. Its acidity^54,55^, and chemical composition^55,59^ affect the kinetics of the maturation stage, as reflected in the resultant performance of a GIC. When compared with artificial saliva, in deionized and/or distilled water, greater fluoride release^59–61^, and mechanical properties^54^, are obtained. This effect results from the inhibitive effect of calcium fluoride deposits^60,61^, which tend to form at the surface of a GIC immersed in an artificial saliva solution^60^. However, no differences are seen in functional properties between the GIC samples stored in deionized or potable water^62^. Nevertheless, most of the available data on maturation processes of GICs concern only early stages of the cements evolution^26^ e.g. as evidenced by changes in strength^26^.

Strength (compression, diagonal extension, and/or biaxial flexure) of modern and experimental GICs has been extensively investigated in the “as-formed” state, e.g.^24,63–65^. There is much less information on how these properties change with aging^64,65^. In some studies, mechanical fatigue resistance of GICs was discussed^66–68^. Nonetheless, little information can be found on the mechanical in vivo behaviour of GICs, as well as their response to in vitro hydrothermal fatigue or the hydrothermal-mechanical cyclic loading.

It has been suggested that modern GICs behave the same way as traditional cements^49^, reaching the highest strength within a month^26,69,70^. This agrees with reports for modern formulations aged in constant temperature and humidity^64,65,70^. High compressive strength should be beneficial in terms of resistance to wear. However, Mahmood et al.^49^ reported that the time evolution of the compressive strength of modern GICs depends on a number of processes which may either positive or negative effect on it. Depending on the relative contribution of these processes, overall strength might undergo changes resisting a simple explanation in particular in the case GICs subjected to thermocycling (Fig. 3).

The compression strength of all tested samples, despite their conditioning method, was over 100 MPa (Figs. 3,  S3–S5, Supplementary Information). This is significant from the view of clinical applications of GICs, as according to international standards^34^, compressive strength of 100 MPa or above is required. Our results indicate that hydrothermal fatigue reduces strength of KU and increases of KME. In the case of RSC a slight reduction of compressive strength is statistically insignificant (Fig. 3). It should be noted that similar divergence in changes of the strength has been reported in^24^. An increase in compression strength was found for Fuji IX, another conventional glass-ionomer cement, while for KME, a decrease was reported after hydrothermal aging, however, under 5000 thermal cycles, what equals ¼ of our aging time.

It was previously claimed that decrease of strength in time is typical for acrylic-maleic acid copolymer glass-ionomers^26^. It is attributed to the fact that acrylic-maleic acid copolymer is characterized by a higher cross-link density than polyacrylic acid, what in the long run makes the cements more brittle compared to the early formulations^71^. However, in our study, all tested cements were acrylic-maleic acid copolymer-based. Moreover, the worst outcome was achieved for the cement in which the polyacrylic acid was not used (KU, Supplementary Table S1). On the other hand, it was found that in higher tartaric-acid GICs, an increased crosslinking density is found due to the action of strongly crosslinking ions, i.e. Al^3+^^49^. This would explain a far higher median compressive strength of the reference RSC and KU samples in comparison with KME. However, in both RSC and KU, after aging, a reduction in both compressive strength (Fig. 2) and surface microhardness (Fig. 3) was found. This finding corresponds with results obtained by Alrahlah^72^, who reported statistically significant reduction in strength and surface hardness of KU subjected to thermocycling. However, the damaging effect of temperature alterations was seen after only 5000 thermal cycles^72^.

Hardness is one of the properties affecting tribological performance of GICs^50^. Time evolution of hardness of GICs was discussed, e.g. in^51,73^. Nevertheless, there is still ongoing controversy with regard to the effect of long-term hardness on GICs. While conventional GICs stored in water tend to increase their hardness^49,51,65,73–75^, for some new compositions, inverse relationships are reported^44,49,50^. Yet, there are factors that make challenging a direct comparison of the obtained values in different reports^36^. For example, in contrast to our study, the vast majority of the available literature data concerns specimens aged at constant temperatures. Indeed, for the reference samples, we obtained similar hardness as presented in the previously published data (e.g. KME:^75–77^; KU:^65,78^, and RSC:^65^). Thus, one may explain the increase in post-fatigue hardness presented in Fig. 2 as related to ambient temperature aging.

It occurred that for KME, irrespective of the applied aging protocol, an increase in hardness was found (35% with reference to the control samples, Fig. 2). This agrees with the results of Brito et al.^79^, who reported hardness of KME GIC after 24 h, 30 days and 180 days of storage in distilled water. However, the increase measured in our study was not so spectacular. On the other hand, after hydrothermal aging, for RSC and KU, a substantial decrease in microhardness was found (approx. 18% for KU and 5% for RSC—see Fig. 2). A decrease in hardness of KU subjected to thermocycling was found also in^72^. On the other hand, an increase in hardness of both KU and RSC was found after 7 and 180 days of aging^65^.

In the polyacid-salt matrix of the GIC kit, water exists in two forms: free water particles, which can be removed from the matrix by drying, and bound water particles, which are chemically locked in the matrix^80^. Since water plays an important role during cement maturation and ion diffusion^4^, properties of GICs are susceptible to both water uptake, as well as dehydration^81,82^. Moisture contamination reduces the mechanical strength of the restoration and its abrasion resistance, while dehydration causes cracks on the surface of the restorations^82^. It is believed that for a long exposure time, GIC softening phenomena caused by water occur in near-to-surface areas only, not affecting the bulk^50^. However, with regard to studies by De Moor et al.^50^, it could be shown that decrease in surface hardness of samples stored in aqueous media may be attributed to a long-term secondary setting reaction. In this reaction, degradation of glass particles is caused by leaching of siliceous species to form silica gel matrix. This corroborates with the recent studies where the proton^83^ and aqueous polyacrylic acid^84^ mobility within GICs were discussed. According to Berg et al.^84^, during the maturation stage of a GIC, loose water can become bound, becoming a part of the cement structure, or remain unbound. For some GICs, with aging, the porosity of the material changes^83^, and the volume fraction of the bound water^83^. However, it was presented that GICs tend to increase the content of unbound water in time^80^. While we observed a great contrast in mechanical and tribological performance between the three tested GICs (Figs. 2, 3, and 5) subjected to thermocycling, further research on water mobility is needed to explain quantitively changes in their strength.

Surface roughness of the glass-ionomer cements is affected by numerous factors, including, but not limited to: matrix characteristics, size and ratio of glass particles, entrapped air bubbles, and others^85,86^. It was previously reported that in an oral environment, bacterial colonization, plaque formation, and its maturation significantly increase when Ra exceeds 0.2 µm^87^. However, in the cited work^87^, ceramic and metallic implant materials were considered. In our study, all tested GICs were characterized by a much greater roughness (Fig. 4), irrespective of the conditioning methods. Nevertheless, the reported values of Ra were still below the tongue limit of roughness distinction, equal to 0.5 µm^88^. In clinical applications, the increased surface roughness of a dental restoration accounts for, among others, plaque accumulation, secondary caries, and loss of aesthetic properties of a material^89^. Rough surfaces promote proliferation in particular of the bacteria responsible for development of caries (*Streptococcus mutans* and *Lactobacillus spp.*) and periodontitis (*Porphyromonas gingivalis* and *Actinobacillosis actinomicetemcomitans*)^90^. However, this effect can at least partially be compensated by the fluoride release.

For in vitro wear testing of restorative materials, various techniques and experimental setups are used^10,12,91^, including, but not limited to: Academisch Centrum for Tandheelkunde Amsterdam (ACTA) wear machine^10,91^, OHSU machine^12^, the Alabama machine^12^, the Dento-Munch Robo-Simulator^12^, and others. While the in vitro test rigs are characterized by a remarkable testing efficacy and control over experiment parameters, the great variability in the applied methodologies and operational concepts make it challenging to compare findings obtained by the different authors. Due to this, the in vitro testing is limited mostly to exploration of trends in tribological behaviours of dental restoratives.

Although a limited number of reports on in vitro testing of tribological performance of matured glass-ionomer cements are available, irrespective of the applied testing protocol and used wear measurement methods, for GICs aged in water under constant temperatures, substantial reduction in wear with time is reported^27,49,92^. This trend tends to reverse if samples are subjected to thermocycling^93^. However, in this study, we report that reaction to thermocycling depends on cement composition (Fig. 5). Stable tribological performance was observed in RSC, as proven by the frictional response (Supplementary Fig. S6), wear (Fig. 5), and morphology of the wear track (Supplementary Fig. S8). The surface softening phenomenon (Fig. 2), as well as post-fatigue reduction in stiffness of this GIC (Supplementary Fig. S3), did not affect its long-term wear resistance. On the other hand, even though surface hardness (Fig. 2) and compressive strength (Fig. 3) of KU were deteriorated by the hydrothermal aging, the cement wear resistance improved (Fig. 5). Also, out of all tested GICs, in RSC only, the same dominant wear mechanisms were identified for samples prior to and after thermocycling (Supplementary Fig. S8). Surprisingly, the worst long-term tribological performance was observed in the case of samples made of KME, which exhibited high hardness (Fig. 2) and compressive strength (Fig. 3). Finally, it should be noted that in comparison with the reference, in the case of thermocycled KU_TC and KME_TC, size of the wear debris produced as a result of friction decreased from micrometric to submicrometric and nanometric scales (Figs. 7 and 8). The effect of such wear products on the oral tissues is yet unknown.

With regard to statistical analysis of the presented data, one has to be aware of the limitations of the study, in particular the sample sizes (n = 5) in compression strength, COF, and wear measurements. In general, the small sample sizes may subject to type II error, that is, failure to reject the false *H*_0_ hypothesis. In particular the non-parametric tests may be underpowered to detect this. Therefore, further studies are advised.

The findings presented here provide new insights on the hydrothermal phenomena influence on the in vitro performance of GICs. However, one should be aware that this is a relatively new field of investigations and available data on the GICs subjected to thermocycling, in particular their frictional and wear behaviour, are scarce. To our knowledge, this is a first work in which the influence of hydrothermal fatigue on the both clinically relevant mechanical and tribological properties are reported.

## Conclusions

Based on the presented findings, the following conclusions are put forward:

1. In contrast to literature data concerning cements aged at constant temperatures, hardness, compressive strength, and wear resistance do not show a consistent increase with time when exposed to temperature cycles.
2. In the case of samples subjected to hydrothermal fatigue, a substantial decrease in the size of wear products, from micrometric to submicrometric and nanometric scales, was found.

The effect of glass-ionomer cement wear micro- and nano-debris on human oral cavity tissues should be further studied.

## Supplementary Information

Supplementary Information.

## Data Availability

The datasets generated during and/or analyzed during the current study are available from the corresponding author on reasonable request.
