# Supplementary material for: The influence of hydrothermal fatigue on the clinically relevant functional properties of conventional glass-ionomer cements
Source: Sci Rep. 2023 May 30;13:8738. doi: 10.1038/s41598-023-35880-4 (PMC10229661; doi:10.1038/s41598-023-35880-4)
Supplement: Supplementary file 1 — Supplementary Information. [file 41598_2023_35880_MOESM1_ESM.docx]

**Supplementary Information**

**The Influence of Hydrothermal Fatigue on the Clinically Relevant Functional Properties of Conventional Glass-ionomer Cements**

Magdalena Łępicka^a*^, Agata Maria Niewczas^b^, Magdalena Urszula Rodziewicz^a^, Konrad Pikuła^b^, Paweł Kordos^c^, Tomasz Gredes^d,e^, Krzysztof Jan Kurzydłowski^a^

^a^Institute of Mechanical Engineering, Faculty of Mechanical Engineering, Bialystok University of Technology, Wiejska 45C, 15-352, Bialystok, Poland

^b^Medical University of Lublin, Department of Conservative Dentistry with Endodontics,
W. Chodzki 6, 20-093, Lublin, Poland

^c^Lublin University of Technology, Institute of Transport, Combustion Engines and Ecology, Nadbystrzycka 36, 20-618, Lublin, Poland

^d^Technische Universität Dresden, Department of Orthodontics, Carl Gustav Carus Campus,

Fetscherstr.74, 01307, Dresden, Germany

^e^Poznan University of Medical Sciences, Department of Orthodontics and Temporomandibular Disorders, Bukowska 70, 60-812, Poznan, Poland

*corresponding author; e-mail: m.lepicka@pb.edu.pl

Table S1. Chemical compositions of the glass-ionomer cements included in the study [1-4]

| **Material Ingredient** | **Ketac Universal** | **Ketac Molar Easymix** | **Riva Self Cure** |
| --- | --- | --- | --- |
| **Liquid** | | | |
| **Acrylic acid-maleic acid co-polymer** | 30 – 50 % | 25 – 40 % | 20 – 30 % |
| **Tartaric acid** | 1 – 10 % | 5 – 10 % | 10 – 15 % |
| **Powder** | | | |
| **Oxide glass chemicals** | > 95 % | 85 – 95 % | 90 – 95 % |
| **Polyacrylic acid** | - | 5 – 15 % | 5 – 10 % |

**References:**

1. 3M ESPE Ketac Universal Safety Data Sheet. 2023 [cited 2023 6th Feb 2023]; Available from: https://multimedia.3m.com/mws/mediawebserver?mwsId=TTTTTV7o0_VyM9Apnyuw5ygCowvaldv_IwVAMYuEvTTTTTT--.

2. 3M ESPE Ketac Molar Easymix Safety Data Sheet. 2023 [cited 2023 6th Feb 2023]; Available from: https://multimedia.3m.com/mws/mediawebserver?mwsId=SSSSSuUn_zu8l9Ul4Yt9ox_9lvVsj17zHvu9lx_D7SSSSSS--.

3. Riva Self Cure (powder) Safety Data Sheet. 2023 [cited 2023 6th Feb 2023]; Available from: https://www.sdi.com.au/pdfs/sds/pl-pl/riva%20self%20cure%20powder_sdi_sds_pl-pl.pdf.

4. Riva Self Cure (liquid) Safety Data Sheet. 2023 [cited 2023 6th Feb 2023]; Available from: https://www.sdi.com.au/wp-content/uploads/SDS/SDS_Pol_PL/Riva_Self_Cure_Liquid_Pol.pdf.

**
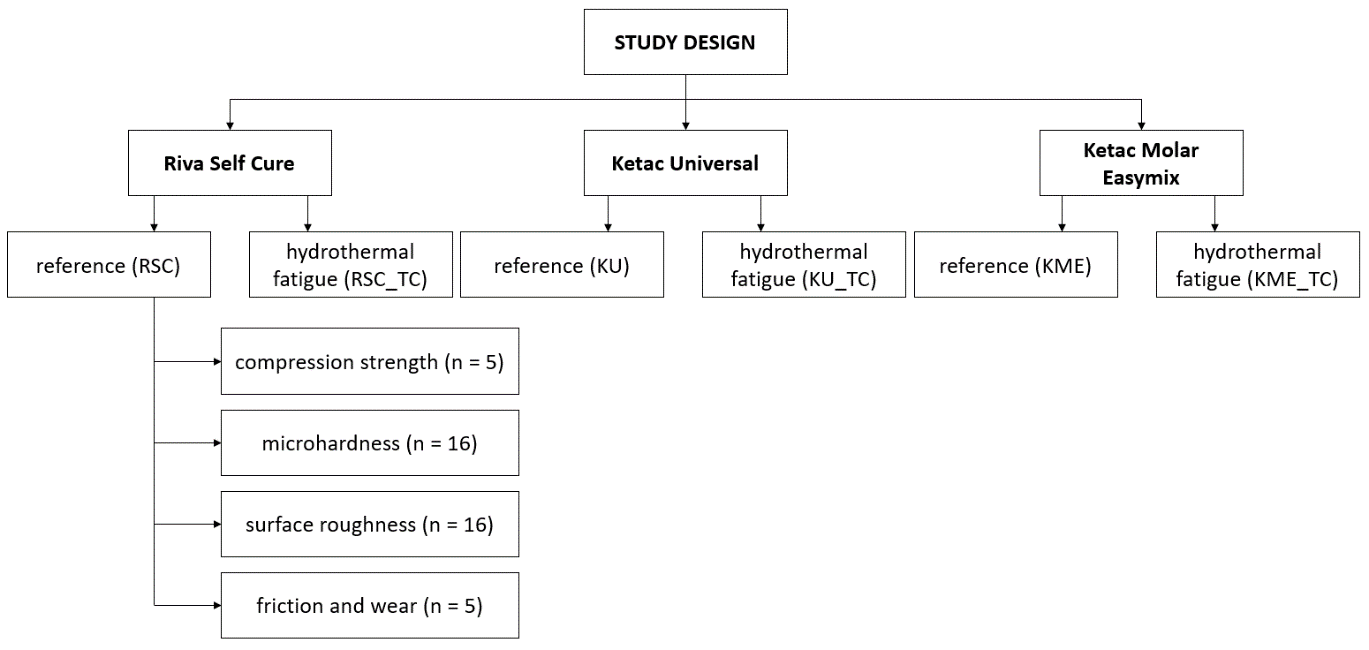
**

Figure S1. Schematic representation of the study design presenting an example of one group workflow


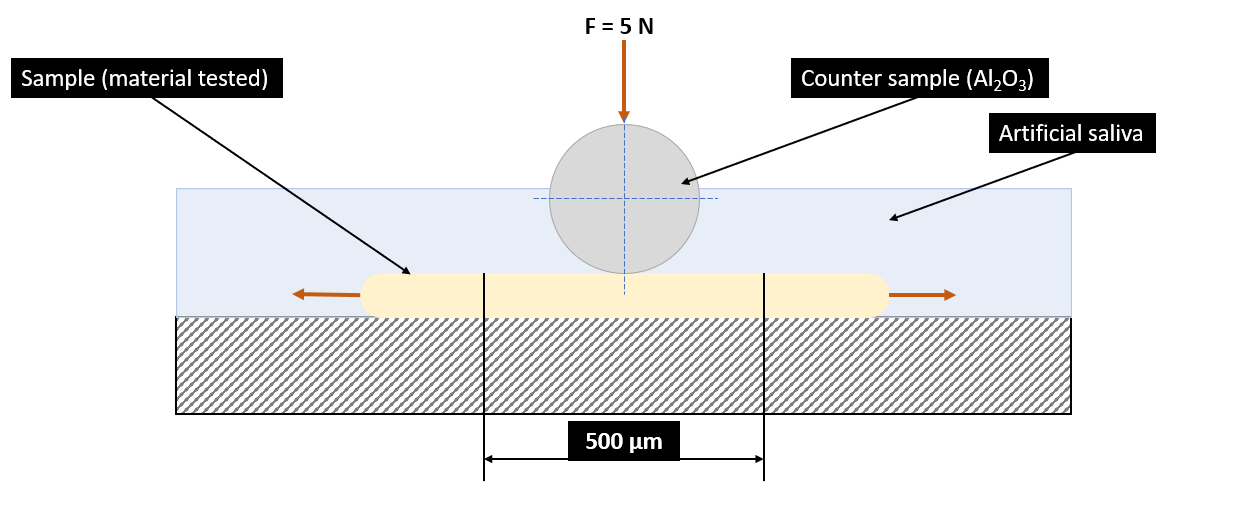


Figure S2. Schematic representation of the friction pair


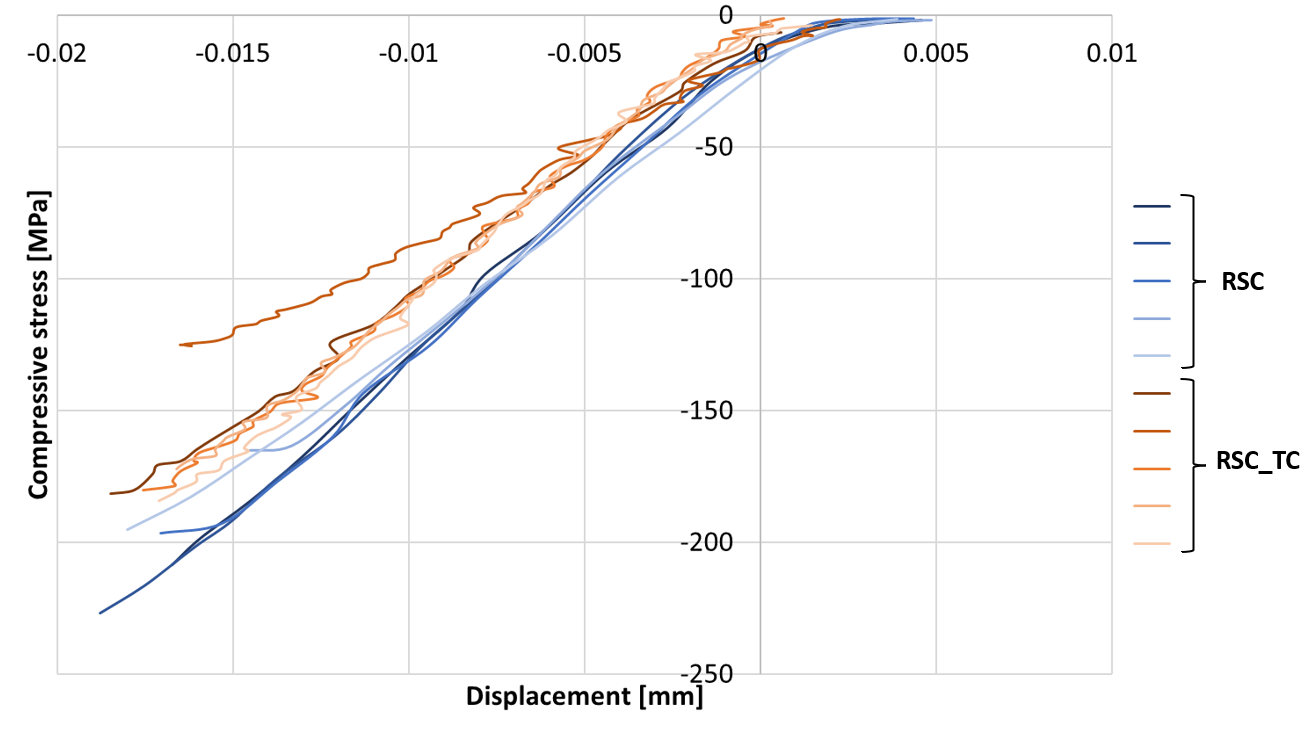


Figure S3. The compressive stress – displacement curves, acquired for the reference and fatigued RSC samples.


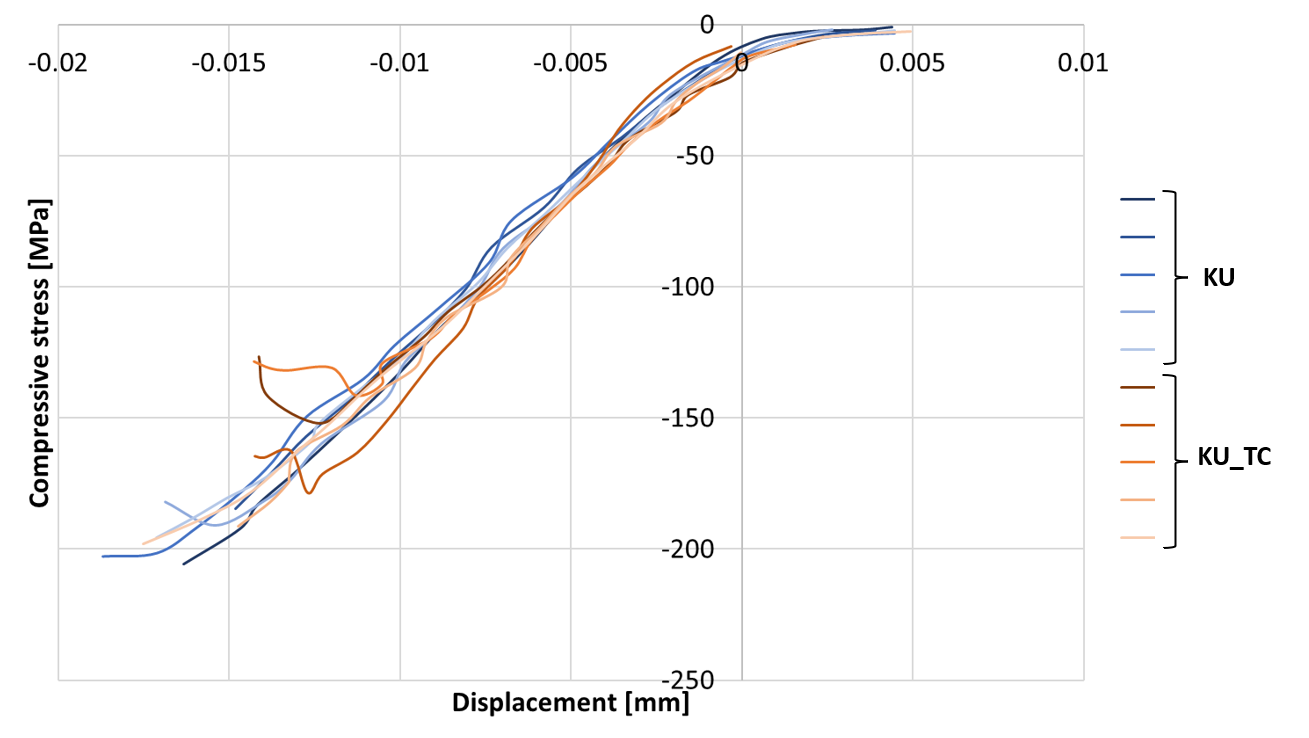


Figure S4. The compressive stress – displacement curves, acquired for the reference and fatigued KU samples.


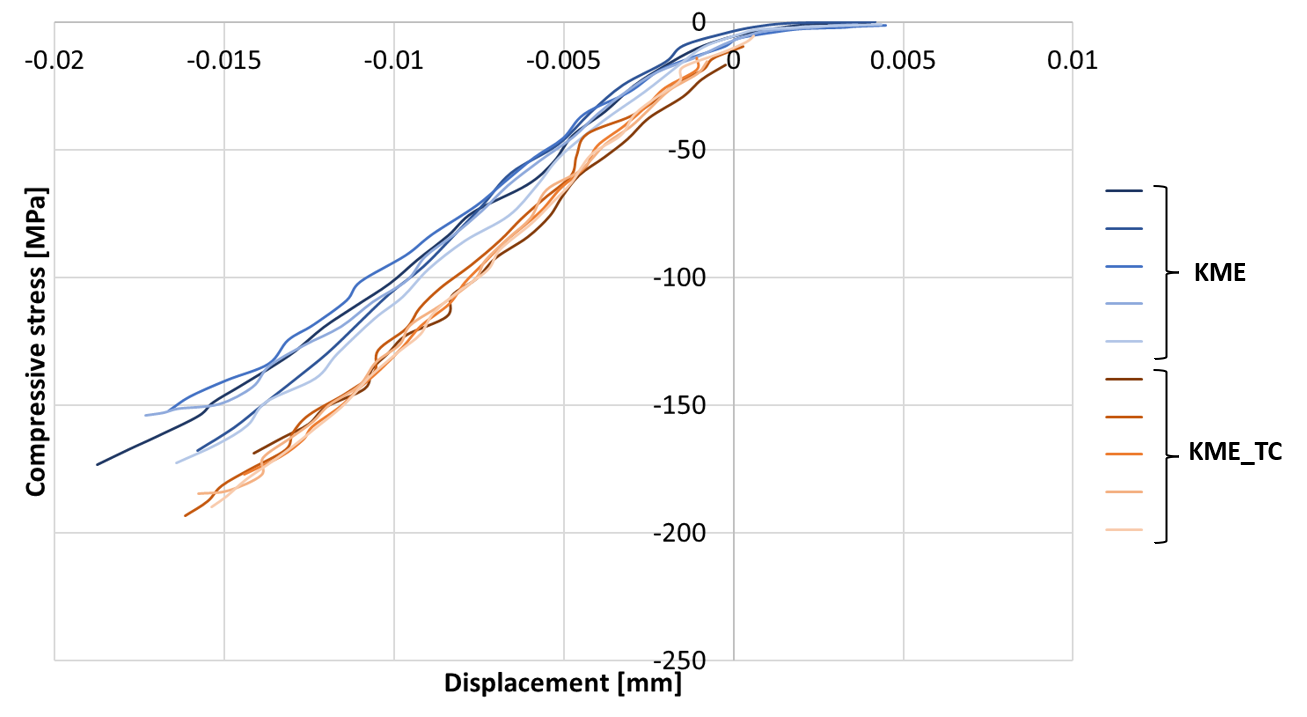


Figure S5. The compressive stress – displacement curves, acquired for the reference and fatigued KME samples.


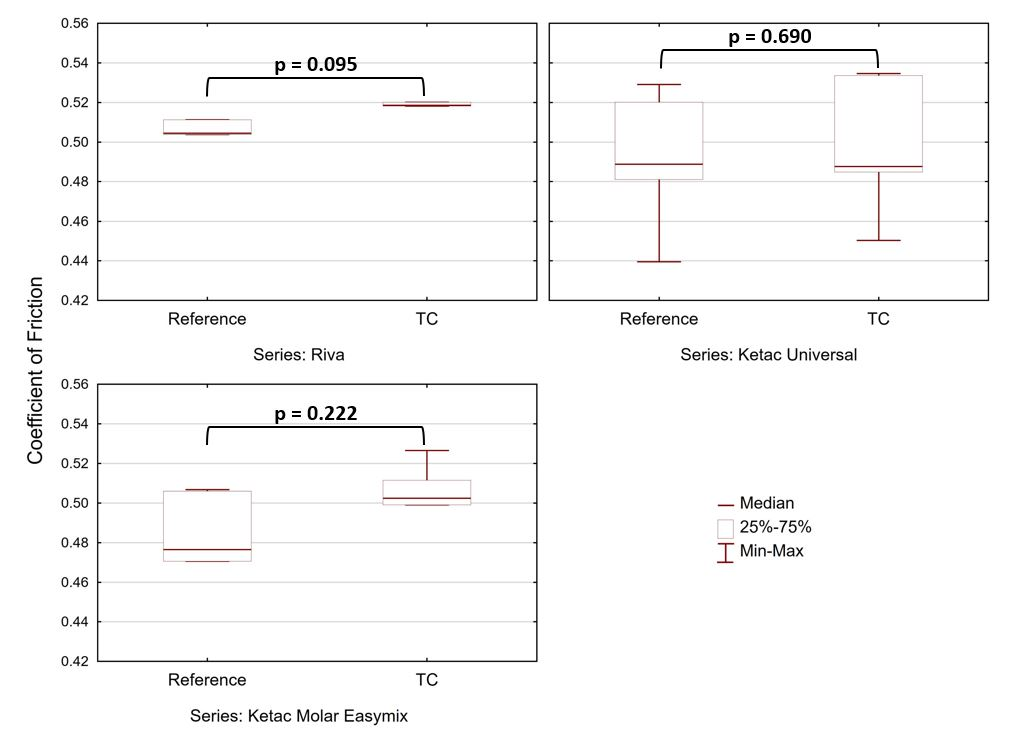


Figure S6. Average COF observed for both reference and thermocycled samples
(U Mann-Whitney, p < 0.05).


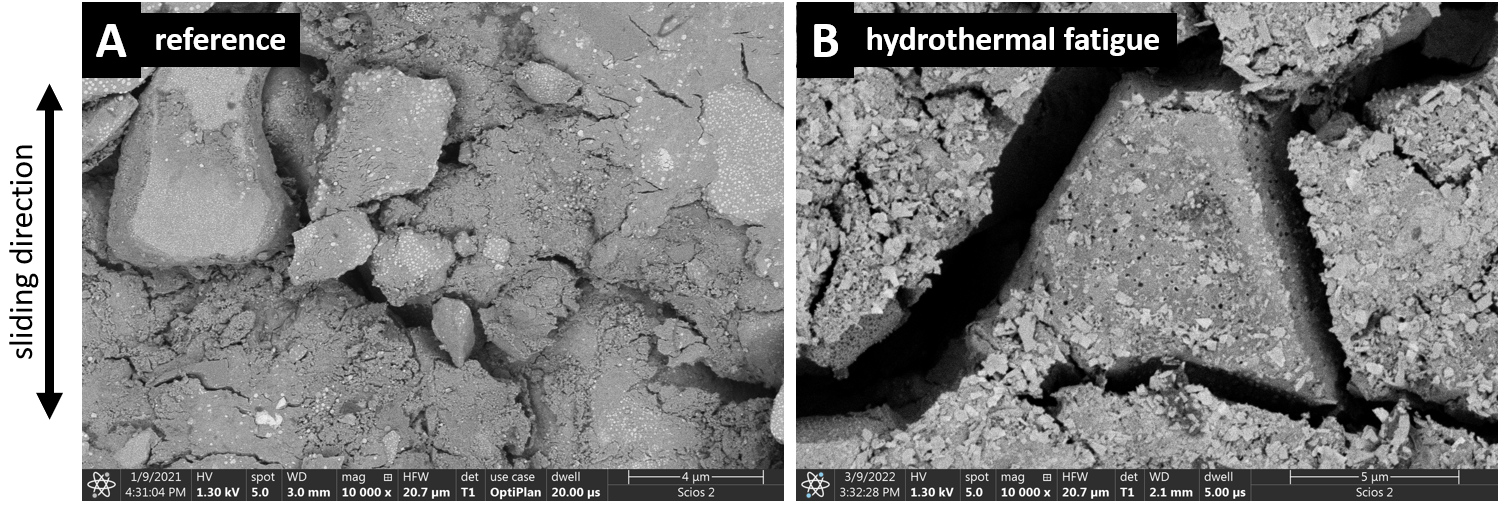


Figure S7. High magnification images of the wear tracks obtained for KME samples: (A) reference, and (B) sample subjected to hydrothermal fatigue. SEM BSE images.


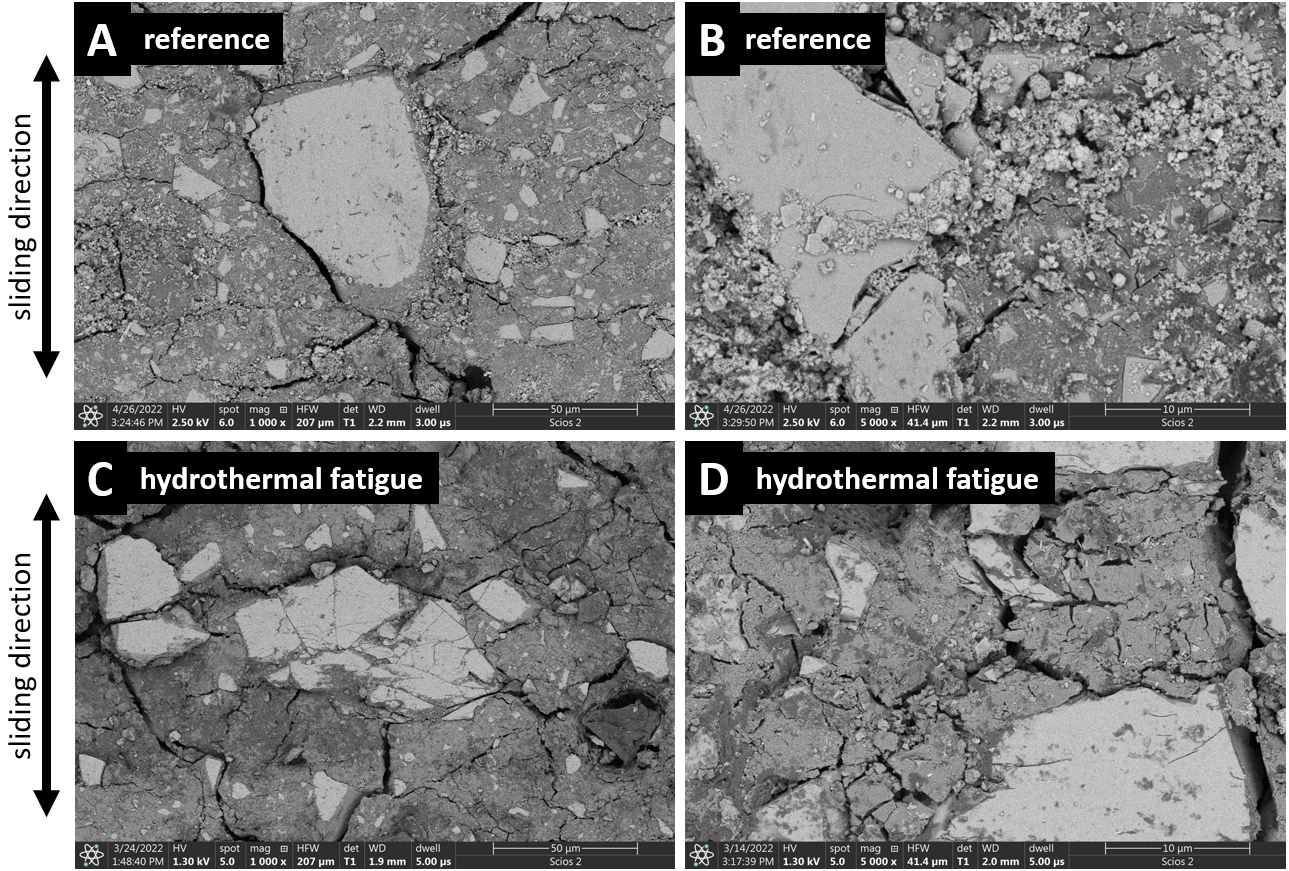


Figure S8. SEM BSE images of the wear tracks obtained for RSC samples: (A) and (B) reference, (C) and (D) sample subjected to hydrothermal fatigue.
